# Synthesis, Physicochemical Characteristics, and Biocompatibility of Multi-Component Collagen-Based Hydrogels Developed by E-Beam Irradiation

**DOI:** 10.3390/jfb14090454

**Published:** 2023-09-01

**Authors:** Maria Demeter, Andreea Mariana Negrescu, Ion Calina, Anca Scarisoreanu, Mădălina Albu Kaya, Marin Micutz, Marius Dumitru, Anisoara Cimpean

**Affiliations:** 1 National Institute for Lasers, Plasma and Radiation Physics (INFLPR), Atomiştilor 409, 077125 Măgurele, Romania; maria.demeter@inflpr.ro (M.D.); marius.dumitru@inflpr.ro (M.D.); 2 Department of Biochemistry and Molecular Biology, Faculty of Biology, University of Bucharest, 050095 Bucharest, Romania; andreea.ne94@gmail.com (A.M.N.); anisoara.cimpean@bio.unibuc.ro (A.C.); 3 Department of Collagen, Division Leather and Footwear Research Institute, National Research and Development Institute for Textiles and Leather (INCDTP), 93 Ion Minulescu Str., 031215 Bucharest, Romania; albu_mada@yahoo.com; 4 Department of Physical Chemistry, University of Bucharest, 4-12 Regina Elisabeta Blvd., 030018 Bucharest, Romania; marin.micut@chimie.unibuc.ro

**Keywords:** collagen, carboxymethylcellulose, e-beam cross-linking, hydrogel, in vitro biological characterization

## Abstract

Herein, three different recipes of multi-component hydrogels were synthesized by e-beam irradiation. These hydrogels were obtained from aqueous polymer mixtures in which different proportions of bovine collagen gel, sodium carboxymethylcellulose (CMC), poly(vinylpyrrolidone), chitosan, and poly(ethylene oxide) were used. The cross-linking reaction was carried out exclusively by e-beam cross-linking at 25 kGy, a dose of irradiation sufficient both to complete the cross-linking reaction and effective for hydrogel sterilization. The hydrogels developed in this study were tested in terms of physical and chemical stability, mechanical, structural, morphological, and biological properties. They are transparent, maintain their structure, are non-adhesive when handling, and most importantly, especially from the application point of view, have an elastic structure. Likewise, these hydrogels possessed different swelling degrees and expressed rheological behavior characteristic of soft solids with permanent macromolecular network. Morphologically, collagen- and CMC based-hydrogels showed porous structures with homogeneously distributed pores assuring a good loading capacity with drugs. These hydrogels were investigated by indirect and direct contact studies with Vero cell line (CCL-81™, ATCC), demonstrating that they are well tolerated by normal cells and, therefore, showed promising potential for further use in the development of drug delivery systems based on hydrogels.

## 1. Introduction

Nowadays, thanks to scientific research, a multitude of alternatives are available for the treatment of various diseases through local and targeted therapy. However, in current medical practice, treatment pathways are still mainly based on the application of oral or parenteral medication. This approach is well known for inducing serious adverse effects on the patient, mainly by destroying healthy cells in the vicinity of the diseased tissue, as happens in the case of cancerous tumors. In this context, it is currently a continuous challenge to develop new materials for the local or targeted treatment of diseases [1]. Current scientific developments demonstrate the effectiveness of local or targeted drug delivery systems (DDS) using hydrogels. For economic and technical reasons, hydrogels represent a safer, cleaner, and non-toxic way to develop DDS [2]. Hydrogels are the most suitable “vehicles” for the local administration of drugs because they allow drug delivery without causing adverse side effects. The widespread use of hydrogels for DDS development is mainly attributed to their excellent properties such as biocompatibility, biodegradability, increased drug loading capacity, and controlled drug release. In addition, hydrogels are generating more interest because their mechanical properties can be easily tailored to mimic the extracellular matrix of most tissues [3].

Hydrogels designed as DDS must meet most of the following properties: be biocompatible, be mechanically resistant, and have a controllable rate of absorption and degradation in physiological environments. They must also be permeable enough to support cell viability and proliferation and facilitate drug release to ensure the desired therapeutic effect. Another parameter that defines the performance of a hydrogel is the degree of cross-linking, a direct indicator of the 3D molecular structure. Furthermore, taking into account the hydrogel network parameters as the polymer volume fraction in the swollen state (υ_2,s_), the average molecular mass between two successive cross-linking points (M_C_), and the mesh size corresponding to the polymer network (ξ), any scientific approach regarding the development of hydrogels as DDS must include the above quality requirements that a hydrogel must meet [4].

An important advantage of hydrogels, both from an economic and technical point of view, is related to the method of obtaining them. Among the different methods of obtaining hydrogels for the development of DDS, cross-linking with ionizing radiation is a fast and ecological method which is carried out at low temperatures, without the addition of catalysts or chemical initiators, and without using complex synthesis strategies. At the same time, the hydrogel is sterilized, a mandatory requirement for any medical product [5].

Among the polymers used in the manufacture of hydrogels, biopolymers are the most common choice due to their excellent properties such as affordable cost, increased biocompatibility and biodegradability, adequate mechanical properties and most importantly, increased ability to absorb fluids. However, despite the excellent biological properties, the production of the hydrogel based only on natural polymers leads to obtaining sticky, soft, and easily broken hydrogels, which cannot be used in biomedical applications due to their poor mechanical properties. Therefore, a mixture of synthetic and natural polymers can lead to the synthesis of hydrogels with a suitable chemical and mechanical structure for use in biomedical applications [6].

Among the natural polymers, collagen and sodium carboxymethylcellulose (CMC) are increasingly used in classical chemical synthesis for the development of hydrogels intended for DDS applications or as dressings for a rapid wound healing process. Collagen is a biodegradable and biocompatible protein produced by fibroblasts, which is found in the connective tissue and is similar to human skin from a compositional and structural point of view [7]. Besides the fact that it provides structural support, it has a role in cell proliferation, differentiation, and migration, in absorbing the exudate and accelerating the natural wound-healing process [8]. Due to its hydrophilic character, CMC has a high affinity for water and excellent compatibility with the human skin, ensuring an optimal moist environment at the wound site, and contributing to the re-epithelialization process [9].

Our research group has extensive long-term experience in obtaining biomedical hydrogels employing e-beam cross-linking. According to works published by our group, the advantages of producing hydrogels using e-beam synthesis, as well as the scientific principles underlying this method, have been widely discussed [10,11,12]. Tichý et al. evaluated the effects of the moist heat sterilization process on the rheological and structural properties of CMC hydrogels [13]. Wang et al. prepared a CMC/poly(vinylpyrrolidone) (PVP) hydrogel with good mechanical properties and swelling ability for wound dressing applications using γ-irradiation [14]. PVP/poly(ethylene glycol) (PEG) hydrogels were synthesized by e-beam irradiation and it has been observed that they have enough strength and can be used as a barrier against microbes; moreover, the irradiation dose had a significant role in the mechanical properties’ improvement [15]. PVP/PEG/agar hydrogel wound dressings were developed by γ-irradiation and showed that the addition of PEG improves the elasticity, adhesion, and overall hydrogel quality [16]. Soler et al. produced pilot-scale batches of PVP/PEG/agar dressings, demonstrating that it is possible to achieve both hydrogel cross-linking and its sterilization by γ-irradiation in a single technological step using the dose of 25 kGy [17]. The hydrogel dressings based on poly(ethylene oxide) (PEO) and PVA were synthesized by e-beam irradiation. Hydrogels with high PEO content had increased gel fractions but were brittle. Their mechanical properties were improved due to the addition of PVA and radiation-induced cross-linking. The healing of wounds treated with such hydrogels is rapid, compared to healing in the open air, as shown by in vivo studies performed on marmots [6]. Chitosan (CS)/PVA dressings with hemostatic, biocompatible, and biodegradable properties were obtained using γ-radiation [18]. CS/PVP/agar hydrogel wound dressing was developed by radiation-induced cross-linking. The CS-based hydrogel showed an antimicrobial effect against Gram-positive bacterial strains [19]. CS/gelatin hybrid hydrogels and CS/gelatin hydrogel loaded with silver nanoparticles were produced by γ-radiation cross-linking at ambient temperature. The hydrogels exhibited appropriate mechanical and biological properties as well as long-term antibacterial activity [20,21].

Interestingly, despite intensive research in the field of DDS development based on hydrogels obtained from collagen, CMC, and PVP as main constituents, we have not identified any study related to the development of multi-component hydrogels with a composition based on bovine collagen, CMC, and PVP or collagen, chitosan, and PVP. In addition, no scientific work has been identified that refers to the synthesis of these new hydrogel compositions using e-beam irradiation. The present study aims to develop and demonstrate the efficiency of manufacturing multi-component hydrogels by electron beam irradiation techniques as well as to evince the suitability of the developed structures to be used as targeted and controlled drug delivery platforms employing in vitro cell-based assays.

## 2. Materials and Methods

### 2.1. Materials

Natural polymers: collagen gel (Type I, concentration 1.65%, pH = 3.1), extracted from calfskin, obtained from the National R&D Institute for Textiles and Leather, Collagen Department by previously reported technology [22]; carboxymethylcellulose sodium salt (CMCNa, Mw = 2.5 × 10^5^ g/mol, degree of substitution, DS = 0.77, viscosity 735 cps, 2% in water, 25 °C); chitosan (CS, degree of N-deacylation = 85%, Mw = 1.9–3.1 × 10^5^ g/mol). Synthetic polymers: poly(vinylpyrrolidone), (PVP1300, Mw = 1.3 × 10^6^ g/mol; PVP360, Mw = 3.6 × 10^5^ g/mol); poly(ethylene oxide), (PEO, Mw = 3 × 10^5^ g/mol); poly(ethylene glycol), (PEG, Mw = 2 × 10^3^ g/mol); N, N’-methylene-bis(acrylamide) (MBA, purity 99%, Mw = 154.17 g/mol), sodium hydroxide (NaOH, 99%, ACS reagent), acrylic acid (AA, anhydrous 99%, ρ = 1.05 g/cm^3^, Mw = 72.06 g/mol). All reagents (except collagen gel) were purchased from Sigma-Aldrich (currently Merck KGaA, Darmstadt, Germany) and used without further purification. Deionized water (DI-water) was prepared in the INFLPR laboratory (resistivity 18.2 MΩ, conductivity 0.055 μS).

### 2.2. Synthesis and Formulation of Multi-Component Hydrogels

The multi-component collagen based-hydrogels composed of collagen, chitosan, and CMC as natural moieties, and PVP and PEG/PEO as synthetic backbone, were simultaneously cross-linked and sterilized by e-beam cross-linking as described below.

To shorten the solubilization times of the polymer mixture components, for PD81 pre-hydrogel stock polymer solutions were prepared by magnetic stirring at room temperature (23 ± 0.5 °C). Polymer solutions with concentrations of PVP1300 (20% *w*/*v*), CMCNa (2% *w*/*v*) PEO (3.5% *w*/*v*) were solubilized in DI-water until homogenization. Three distinct polymer mixtures with the following composition, expressed per 100 mL of pre-hydrogel (uncross-linked polymer mixture), were prepared from previously prepared stock solutions as shown in Table 1.

CMC is considered an intelligent cellulose derivative since it shows high sensitivity to pH, ionic-strength variations, and good swelling capability. Besides these properties, it presents excellent biocompatibility and biodegradability and its increased water affinity is due to the highly reactive hydroxyl and carboxyl groups contained in its backbone. Recently, it was demonstrated that CMC presents interesting abilities to form polymer-drug conjugates with drugs that are poorly soluble in water [23]. The polymer-drug conjugation formed by amide covalent bonds offers a new approach regarding the development of topical drug delivery systems. Despite this advantage, CMC along with other natural polymers, such as collagen and chitosan, has low mechanical strength and high biodegradability. For this reason, the association between CMC, collagen, and synthetic polymers such as PVP, which has a low swelling capacity, offers the possibility of obtaining biostable hydrogels due to the affinity of PVP to cross-link easily through irradiation and forming 3D networks that are soft and flexible. Collagen as an ingredient in a multi-component hydrogel can also increase the loading capacity of various bioactive substances, but its major role in this recipe is to facilitate the interaction between cells and the designed hydrogel by increasing cell attachment, proliferation, and differentiation. Polymers such as PEG or PEO have the role of increasing the flexibility and elasticity of the structure of a hydrogel. They contain electron-donating groups that ensure a better binding of bioactive substances but also increase hydrophilicity.

### 2.3. E-Beam Cross-Linking of Multi-Component Hydrogels

After complete homogenization of the polymer mixtures (pre-hydrogel) by magnetic stirring at 23 ± 0.5 °C, the pH of the solutions was gradually adjusted with 1 M NaOH to 7.2–7.4. Before e-beam cross-linking, the pre-hydrogels were centrifuged (500–800 rpm, 10–20 min) and degassed with ultrasound (5–10 °C, 37 Hz, 30 min). The purpose of the mentioned operations was to remove the dissolved oxygen that was introduced into the pre-hydrogel during mixing to avoid the oxidative process that occurs during irradiation. These operations ensure the obtaining of a hydrogel with a uniform and homogeneous surface. Depending on the final shape of the hydrogel and before irradiation, the pre-hydrogel was distributed in sterile plastic containers (d_i_ = 1.2 cm, h = 0.5 cm, V = 0.7 cm^3^) suitable for the final purpose, having the possibility of hermetic closure. For the hydrogel samples to be easy to handle after cross-linking (especially for biological tests), the polymer solutions were cast on cellulose discs with Φ 1.2 cm. To establish the optimal irradiation dose, the polymer mixtures were irradiated with doses between 5–40 kGy at an average absorbed dose rate of 4 kGy/min.

E-beam irradiations were performed using the ALID-7 linear electron accelerator (6 MeV average electron energy) at the Accelerators Laboratory, National R&D Institute for Laser, Plasma, and Radiation Physics. Irradiations were performed under the following conditions: average beam current of 5 μA, filament voltage 12 V, electron pulse duration of 3.75 μs, and electron pulse repetition rate of 53 Hz. Nominal dose, dose distribution, and irradiation geometry as well as absorbed dose measurements were measured using dosimetric systems based on graphite calorimeters and B3 radiochromic films calibrated against reference alanine dosimeters.

### 2.4. Characterization of Multi-Component Hydrogels

#### 2.4.1. Sol-Gel Analysis

The hydrogels were dried in an oven below 30 °C for 72 h (m_0_), then were extracted with DI-water at room temperature for 48 h and further dried in the same temperature conditions for 72 h (m_d_) up to constant weight. The cross-linked content (gel fraction) was determined gravimetrically by using the following Equation [24].

(1) $$\mathrm{GF}\left(\%\right)=\left(\frac{m_{d}}{m_{0}}\right)$$

The main parameters of the radiation cross-linking process as the gelation dose (D_g_); the virtual dose *(*D_v_) and the ratio of radiation yield of scission to radiation yield of cross-linking (p_0_/q_0_ were evaluated with GelSol95 v1.0 software, made at Lodz University of Technology, Division of Applied Radiation Chemistry, Lodz, Poland) based on the Charlesby–Rosiak equation [25].

(2) $$s+\sqrt{s}=\frac{p_{0}}{q_{0}}+\left(2-\frac{p_{0}}{q_{0}}\right)\times \left(\frac{D_{v}+D_{g}}{D_{v}+D}\right)$$

Radiation chemical yields of cross-linking and degradation were calculated using the following Equations [26]:

(3) $$G\left(X\right)=\frac{4.9\times 10^{2}\times c}{M_{C}\times D\times \rho }$$

(4) $$G\left(S\right)=G\left(X\right)\times 2\frac{p_{0}}{q_{0}}$$

where *s* is the sol fraction of polymer, D is the absorbed dose (kGy), G(X) is the radiation chemical yield of cross-linking (mol/J), G(S) is the radiation chemical yield of scission (mol/J), Mc is the average molecular weight between two successive cross-links (kg/mol), c is the polymer concentration (g/L), and ρ is the density of the polymer (kg/m^3^). D_v_ represents the necessary dose to transform the actual sample into a sample of the most probable molecular weight distribution of M_w_*/*Mn = 2 [27].

#### 2.4.2. Swelling Degree

The hydrogel swelling properties were evaluated in DI-water and different pH buffer solutions (citrate (pH 5.4), saline phosphate (PBS, pH 7.4), and bicarbonate (pH 9.4)) at 37 °C using a thermostatic oven. At the established time, the hydrogels were removed from the swelling medium, gently blotted with filter paper to remove the solvent excess on the hydrogel surface, and weighed. The swelling degree (SD) was calculated using the Equation [28].

(5) $$\mathrm{SD}=\frac{\left(m_{s}-m_{0}\right)}{m_{0}}\times 100$$

#### 2.4.3. Moisture Retention Capability and Water Vapor Transmission Rate

The moisture retention capacity (R_h_ (%)) was calculated after cutting each hydrogel pad into 2.5 × 2.5 cm^2^ pieces. They were each placed in a Petri dish and their initial weight (m_0_) was measured at room temperature (25 ± 1 °C). The Petri dishes together with the hydrogels were left in the laboratory under ambient conditions. They were weighed at set time intervals (m_t_). Based on the measurements, R_h_ was determined as the ratio between m_t_ and m_0_ according to Equation (6) [29].

(6) $$R_{h}\left(\%\right)=\frac{m_{t}}{m_{0}}\times 100$$

Water vapor transmission rate permeability (WVTR) was measured using the procedure contained in a monograph of the European Pharmacopoeia. According to the procedure, the weight loss of a bottle with a mouth of 28 mm and containing 8 mL of DI-water was measured. The hydrogel sample, with a diameter of 35 mm, was placed on the mouth of the bottle and this assembly was placed in an oven for 24 h where the temperature was maintained at 37 °C. The WVTR was calculated with the Equation (7) [30]:

(7) $$\mathrm{WVTR}=\frac{\left(m_{0}-m_{t}\right)}{\left(A\times 24\right)}\times 10^{6}{g\;m}^{-2}{\;h}^{-1}$$

where A is the area of the bottle mouth (mm^2^), m_i_ and m_t_ are the weight of the bottle before and after being placed in the oven, respectively.

#### 2.4.4. Network Structural Parameters

Rheological analysis can be used to evaluate the cross-linked network structure of the multi-component hydrogels. The average molecular weight between two successive cross-links ($M_{c}$), cross-link density ($V_{e}$), and mesh size (ξ) of the multi-component hydrogel can be determined using the following Equations [31,32]:

(8) $$M_{C}=\frac{A\rho \mathrm{RT}{\left(\upsilon_{2r}\right)}^{2/3}{\left(\upsilon_{2s}\right)}^{1/3}}{G\text{’}}$$

(9) $$V_{e}=\frac{\rho }{M_{C}}$$

(10) ξ=ν2s−1/3×[Cn(2MCMr)]−12×l

where the factor A for an affine network (A = 1), R is the universal gas constant (8.314 m^3^·Pa/mol·K), T is the absolute experimental temperature (298.15 K), υ_2r_ is the polymer volume fraction after e-beam cross-linking, υ_2S_ is the polymer volume fraction of the cross-linked hydrogel in the swollen state, G′ is the elastic modulus (Pa), C_n_ is the Flory characteristic ratio expressed as the average molecular weight of the polymers: collagen = 9 [33], PVP = 12.3 [34], CS = 32.8 [35], CMC = 10 [36], AA = 6.7 [37], PEG/PEO = 4.0 [27]. M_r_ is the molecular weight of the monomer unit, taken as a weighted average of the molecular weights: collagen = 321.32 g/mol; PVP = 111.14 g/mol [34]; CS = 161.2 g/mol [38]; CMC = 234 g/mol; AA = 72.06 g/mol [37]; PEG/PEO = 44.05 g/mol [39], and l is the carbon–carbon bond length (0.154 nm).

#### 2.4.5. Rheological Analysis

The hydrogels’ rheological properties based on G′ (storage modulus), and G″ (loss modulus) were determined using a Micro Fourier Rheometer MFR 2100 (GBC, Melbourne, Australia).

The rheometer is provided with a temperature control device connected to a Lauda E100 water bath. The rheological measurements were performed by inserting the hydrogel samples between two circular parallel plates. In this configuration, a compressive oscillatory flow is applied under the vertical oscillation of the upper plate. The upper circular plate exerts on the stationary bottom one a force directly sensed by a dedicated load cell, using an oscillatory motion of pseudorandom noise shape consisting of many sinusoids summed over a given frequency range.

All rheological parameters that interested us (G′, G″, complex modulus, complex viscosity, and its components, loss tangent) were obtained by Fourier analysis of the force signal acquired for the 400 discrete frequencies concomitantly. The instrument set-up was as follows: the gap between plates—400 μm, displacement amplitude—0.03 μm (to be in the linear viscoelasticity domain), frequency interval—0.05–20.00 Hz (with an increment of 0.05 Hz), equilibration time—20 min, 30 scans/sample. The rheograms were obtained in the angular frequency range of 1.57–100 rad/s. All rheological measurements were carried out at the ambient temperature of 23.0 ± 0.1 °C. The measurements were performed in triplicate having a relative SD of less than 15%.

#### 2.4.6. Fourier Transform Infrared (FTIR) Analysis

The FTIR spectra of the unirradiated polymeric mixture and cross-linked hydrogel samples were taken with a Spectrum 100 instrument (Perkin Elmer, Waltham, MA, USA) equipped with the diamond crystal. Before the FTIR analysis, the samples were freeze-dried. The FTIR spectra were obtained in ATR mode in the range of 4000–600 cm^−1^ with 50 scans/sample and a resolution of 4 cm^−1^.

#### 2.4.7. Scanning Electron Microscopy (SEM)

The multi-component hydrogel morphology was investigated by using a 20 kV scanning electron microscope (SEM) FEI Inspect S model (FEI Co. Ltd., Hillsboro, OR, USA). Hydrogel samples swollen to equilibrium in DI-water were freeze-dried before SEM analysis. The cross-section of the freeze-dried hydrogels was observed after gold coating. The SEM images were acquired at magnifications of 250×, 500×, and, respectively, 1000×.

### 2.5. In Vitro Biological Characterization

#### 2.5.1. Cell Culture Model and Extraction Medium Preparation

For the in vitro cell-based investigations, commercially available VERO kidney epithelial cells extracted from an African green monkey (American Type Cell Culture, Manassas, VI, USA) were used. The cells were grown in Minimum Essential Medium (MEM, Sigma-Aldrich Co., St. Louis, MO, USA) supplemented with 10% fetal bovine serum (FBS, Life Technologies Corporation, Grand Island, NY, USA) and 1% (*v*/*v*) penicillin (10,000 units/mL)/streptomycin (10 mg/mL) (Sigma-Aldrich Co., St. Louis, MO, USA) at 37 °C in a humidified atmosphere with 5% CO_2_. The standard culture medium was changed every 2 days and when it reached 80% confluency, the cells were trypsinized and seeded either directly onto the surface of the analyzed samples at an initial density of 1.5 × 10^4^ cells/cm^2^ for direct contact studies or on 48-well-plates in the corresponding extraction medium at an initial density of 1 × 10^4^ cells/cm^2^ for indirect contact studies. To perform the indirect contact studies, extracts corresponding to each sample were prepared according to Wallin [40]. In essence, the sterilized hydrogels were incubated for 24 h at 37 °C in standard culture medium free of serum, at a ratio of the surface area to the volume of extractant used of 3 cm^2^/mL. In the end, the resulting extracts were collected and supplemented with 10% FBS for their further use in cell culture-based studies.

#### 2.5.2. Indirect Contact Studies

To investigate the effect of the extraction medium on the cellular behavior, a tetrazolium dye (MTT, (3- (4,5-dimethylthiazol-2-yl) -2,5-diphenyltetrazolium bromide)) colorimetric assay was performed at 24 h and 72 h post-seeding. Briefly, after removing the extraction media, the cells were rinsed with PBS and incubated for 3 h at 37 °C with a MTT solution (1 mg/mL). Ultimately, at the end of the incubation period, the amount of formazan generated by the metabolically active viable cells was measured at 550 nm using a microplate reader (FlexStation 3 microplate reader, Molecular Devices, San Jose, CA, USA). Moreover, the morphological characteristics of the cells were highlighted through microscopical observations at 24 h post-seeding, with representative fields captured using the cellSens Dimension acquisition system Version 4.1 (Den Haag, The Netherlands).

#### 2.5.3. Direct Contact Studies

To observe the possible cytotoxic effects of the newly developed hydrogels, the viability of the Vero cells grown in direct contact with their surfaces was evaluated at 24 h and 72 h post-seeding through a qualitative assay, namely, calcein acetoxymethyl ester (AM)/ethidium homodimer-1 (EthD-1) staining with the help of a LIVE/DEAD Cell Viability/Cytotoxicity Assay Kit (Molecular Probes, Eugen, OR, USA) following the manufacturer’s instruction. Therefore, at the end of the experimental time, the samples were rinsed with PBS and incubated for 10 min at room temperature, in the dark with the staining solution. In the end, the marked cells (live cells—green fluorescence; dead cells—red fluorescence) were visualized with an inverted fluorescence microscope (Olympus IX71, Olympus, Tokyo, Japan) and the representative fields were captured with the cellSens Dimension acquisition system Version 4.1. At the same time points, a quantitative method, the Cell Counting Kit (CCK)-8, was employed to assess the proliferative status of the Vero cells. For this purpose, the medium was removed and in each well fresh culture medium containing 10% CCK-8 reagent was added. After 2 h of incubation at 37 °C, the optical density was measured at 450 nm employing an automatic plate reader (FlexStation 3, Molecular Devices, San Jose, CA, USA).

Additionally, the activity of the lactate dehydrogenase (LDH) released into the culture medium as a marker of cell death and lysis of the plasma membrane was investigated using a cytotoxicity kit (Tox-7, Sigma-Aldrich Co., St. Louis, MO, USA) at 24 h and 72 h post-seeding. The optical density of the resulting product was measured at 490 nm with the help of a microplate reader (FlexStation 3 microplate reader, Molecular Devices, San Jose, CA, USA).

### 2.6. Statistical Analysis

The obtained data were statistically analyzed with the help of the GraphPad software (Version 8, GraphPad, San Diego, CA, USA) using two-way ANOVA with Tukey’s multiple comparison tests. Data are presented as means ± SD (standard deviation) and only *p*-values < 0.05 were considered statistically significant. All physicochemical measurements were carried out in triplicate and the results were given as mean value and standard deviation (SD).

## 3. Results and Discussion

### 3.1. Sol-Gel Analysis

**Figure 1:**
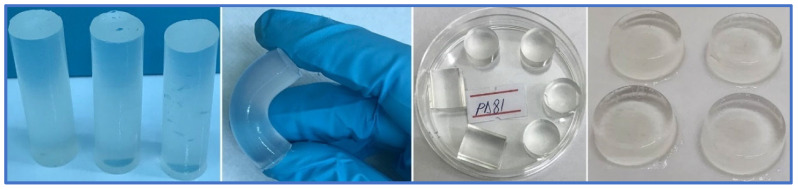
PD81 multi-component hydrogels obtained after e-beam irradiation in different shapes.

To optimize the recipe for obtaining the multi-component hydrogels, the evaluation of the radiation cross-linking process was carried out with the sol-gel analysis by which the following parameters were determined: gel fraction (GF), cross-linking G(X) and scission G(S) yields, and the gelation dose (Dg). Dg is the minimum irradiation dose at which the sol-gel transition occurs. Figure 1 shows the appearance of the multi-component hydrogels obtained after e-beam irradiation in different shapes. It can be seen that all hydrogels are transparent, maintain their structure, are non-adhesive when handling, and most importantly, especially from the point of view of the application, they have an elastic structure.

**Figure 2:**
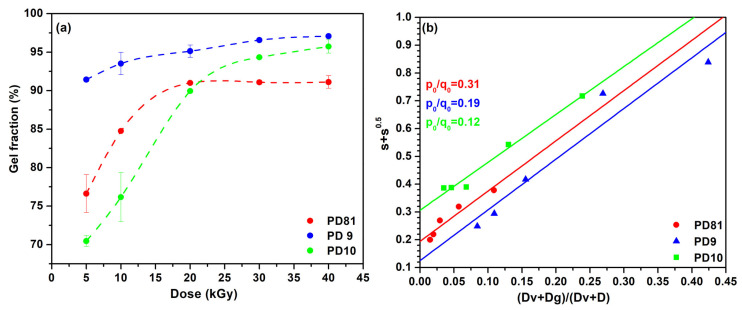
(**a**) Effect of absorbed dose on gel fraction; (**b**) Sol-gel analysis corresponding to the Charlesby–Rosiak equation.

Figure 2a illustrates the gel fraction as a function of the absorbed dose. The gel fraction is increased even at low doses. For example, at 5 kGy it is greater than 70%, a value confirmed for all of the studied hydrogel compositions and which increased with the absorbed dose. The highest GF was obtained for composition PD9, over 90% at 5 kGy. For compositions PD81 and PD10 at absorbed doses above 20 kGy, the GF remains constant and is between 90% and 97%. The PD9 composition, unlike the PD81 and PD10 compositions, cross-links very quickly, this being the direct contribution of the cross-linking agent (MBA), but also of the increased PVP concentration. With the increase in the absorbed dose, no degradative contribution was observed regarding the GF. The radiation-induced cross-linking of biopolymers is generally accompanied by significant degradation of the polymer chain, visible through the decrease of the GF. From Figure 2a, it can also be seen that in the case of compositions PD81 and PD10, in which the concentration of biopolymers (collagen, CMC, or chitosan) is higher, higher irradiation doses are needed to increase the GF above 75%. In the PD81 composition in which no cross-linking agent was added, the cross-linking reaction occurs only due to the contribution of synthetic polymers. In a polymeric system consisting of both synthetic and natural polymers, the degradative effects can compete with the cross-linking ones. In this case, from a practical point of view, it can be done either by increasing the irradiation dose or by using an appropriate concentration of cross-linking agent.

To obtain gels with GF of only 40% by irradiating pure CMC solutions, Fekete et al. used irradiation doses of over 60 kGy. These authors also demonstrated that increased doses of irradiation are detrimental to the cross-linking process [41]. Therefore, the association of biopolymers with synthetic polymers significantly improves the yield of the cross-linking reaction, which is demonstrated in this study.

The cross-linking degree of a polymeric system can also be highlighted by determining the p_0_/q_0_ ratio (degradation vs. cross-linking) according to the Charlesby–Rosiak equations, which quantifies the relationship between radio-induced degradation and cross-linking processes [42,43]. For polymer mixtures that form insoluble fractions as a result of cross-linking induced by irradiation, the ratio p_0_/q_0_ is less than 2, which shows that the cross-linking processes are predominant [19].

From Table 2 and Figure 2b, it can be seen that the ratio p_0_/q_0_ is between 0.12 and 0.31, which according to the previous literature data proves that the cross-linking processes are predominant. The second method of evaluating the irradiation process of the developed hydrogels from aqueous polymer mixtures consists of quantifying the effect of ionizing radiation, both in terms of cross-linking and degradation of polymer chains. For this purpose, the following parameters are frequently used: radiochemical yield of cross-linking G(X), and radiochemical yield of degradation, G(S). To determine G(X), respectively, G(S), it is necessary to know the absorbed dose [44]. Table 3 shows the values of the G(X), respectively, of G(S) depending on the absorbed dose and the composition of the hydrogels. For all hydrogel compositions tested, a general trend of G(X) decreasing with increasing the absorbed dose was observed. At irradiation doses between 5 and 10 kGy, the cross-linking yields increase in the order PD81 < PD9 < PD10, and in the dose range 20–30 kGy, G(X) has approximately constant values.

For all three hydrogel compositions, G(X) > G(S), which shows that cross-linking processes prevailed compared to degradation processes. These results confirm the obtaining of hydrogels with stable structures after irradiation. To obtain a cross-linked hydrogel, but also one sterilized in the same technological step, for the final formulation of the hydrogel it was determined that the optimal irradiation doses are between 20 and 30 kGy.

### 3.2. Swelling Degree

Determination of hydrogel properties such as swelling properties and mechanical properties depending on the environmental variation (temperature, pH) are mandatory methods to evaluate the physical and chemical stability of hydrogels. The swelling degree and the structural stability of the hydrogel are two essential characteristics for their use in applications as a support for controlled drug release.

The multi-component hydrogels formulated according to PD81, PD9, and PD10 recipes showed different degrees of swelling in DI-water and buffer solutions. The PD81 composition shows the highest swelling degree in all media with different pH, except for the pH 9.4 solution. In DI-water, hydrogels have an equilibrium swelling capacity between 1000–3000%, reach equilibrium after 8–10 h after water immersion, and retain their stability and shape for more than 48 h, without dissociating in the swelling medium. The swelling capacity values obtained in the case of the PD81 composition are comparable to hydrogels prepared from collagen, CMC, and silver nanoparticles obtained by cross-linking with glutaraldehyde [9]. The lower swelling capacity of the PD9 hydrogel can be explained by the increase in the cross-linking density, this being a direct consequence of the use of an increased concentration of PVP, but also of the use of MBA as a cross-linking agent.

The swelling capacity in environments with pH between 5.4 to 7.4 is lower, but even in these conditions the hydrogels are stable, do not dissociate in the swelling environment, and retain their superabsorbent characteristics.

In an acidic environment, swelling is reduced due to the protonation of the carboxymethyl and carboxylic groups contained in the hydrogel networks. In a swelling medium of pH 5.4 and due to the pKa 4.3 of carboxyl contained in CMC, the COOH groups are in the ionized form, i.e., in a deprotonated state. As for the NH_2_ groups, at pH 5.4 they are in a protonated state, which facilitates the ionic interaction between COO^−^ and ${\mathrm{NH}}_{3}^{+}$, giving a more compact hydrogel. On the contrary, in a more alkaline pH, the amino groups are not protonated, which facilitates electrostatic repulsions. This behavior was also observed in the case of gels based on CMC and acrylic acid [41] and carboxymethylchitosan hydrogels [45].

**Figure 3:**
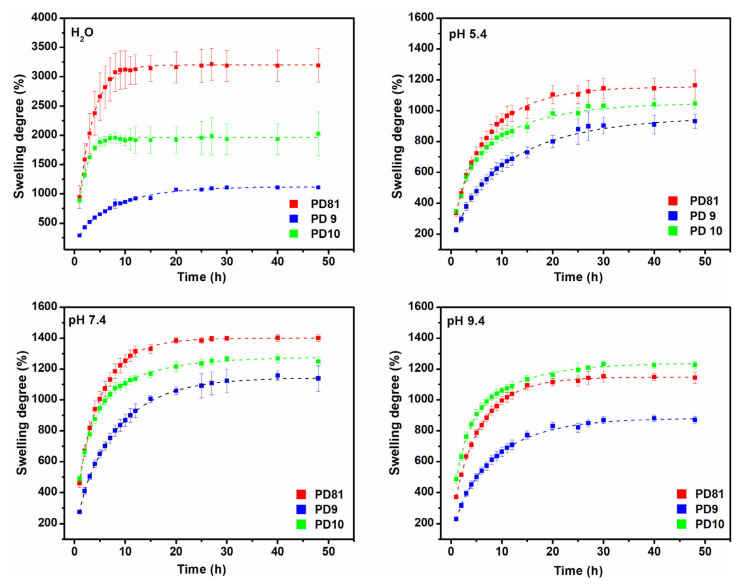
The multi-component hydrogels’ swelling degree in different pHs at the temperature of 37 °C (presented as mean ± 3 replicates).

From Figure 3 it can also be observed that in a neutral environment, the swelling degree is higher than the corresponding one in acidic and basic environments. This results from the activation of the deprotonation mechanisms of proton donor groups (${\mathrm{NH}}_{3}^{+}$, COOH, OH), as a result of the disintegration of inter-molecular or intramolecular cross-linking points in acidic and basic environments, which ultimately leads to an increase in the mesh size of the hydrogels’ network, so there is an increase in fluid absorption in the macromolecular network of the hydrogel [46]. Moreover, increased absorption in an alkaline environment is beneficial in the case of using hydrogels as dressings for chronic wounds. The greater ability to swell in the case of the PD81 composition is mainly due to the low concentration of polymers, but also to the increased concentration of hydrophilic components such as collagen and CMC.

### 3.3. Network Parameters

In biomedical applications aimed at cell regeneration, it is of great interest to investigate the hydrogel network characteristics. For this reason, in the case of the development of hydrogels by e-beam cross-linking, a particular interest consists of establishing a relationship between the hydrogel mesh size and the effect of irradiation on its structure [47].

In the following, by correlating the rheological properties and the degree of cross-linking with the specific structural parameters of the hydrogels, the cross-linked structure of the hydrogels was characterized. The network parameters specific to a hydrogel (Mc, Ve, and ξ) were determined from rheological analysis using the rubber elasticity theory [48]. Table 4 shows the structural parameters of the multi-component hydrogels obtained by irradiation with a dose of 25 kGy. The network parameters Mc, ξ, and Ve varied according to the composition of the hydrogels as follows: the PD81 composition has the highest cross-linking density (Ve = 1.17 mol/m^3^), which shows the formation of a hydrogel with a dense structure and high stability.

Compared to the PD81 and PD9 hydrogels, the PD10 hydrogel shows a significant decrease in the cross-linking density, also correlated with the decrease in the elastic modulus. It can be observed that the mesh size of the PD9 hydrogel network decreases, which means the formation of a network with several cross-linking points formed inside it. This is mainly due to the addition of a cross-linking agent, which of course increases the yield of the cross-linking reaction, but decreases the ability of the polymer network to incorporate fluids into its network. Thus, this composition presented a pronounced elastic character. Otherwise, the PD10 composition is characterized by increased mesh size and a moderate absorption power compared to PD81.

**Figure 4:**
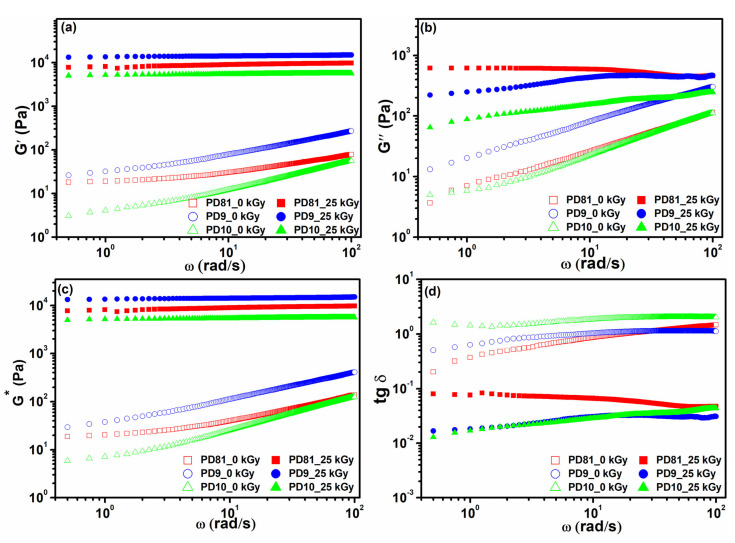
Variation of elastic G′ (**a**), viscosity G″ (**b**), complex G* (**c**) moduli, and loss tangent as a function of angular frequency (**d**).

Hydrogels PD81 and PD10 were prepared using PVP with a high molecular mass, unlike the composition PD9, for which PVP with a lower molecular mass was used. It is obvious that by using a PVP with a lower molecular weight and a concentration of 7%, mechanically resistant hydrogels are obtained. In this case, we appreciate that if the macromolecular chains are shorter, the mobility of the radicals resulting from the radiolysis process is facilitated, naturally leading to the formation of more chemical bonds in the hydrogel network. According to Fekete et al. [41], an improved cross-linking has been observed in the case of CMC/acrylic acid hydrogels produced by gamma-radiation synthesis. They assumed that due to the higher mobility of acrylic acid molecules than CMC macromolecules, the cross-linking was significantly improved. Another factor has been attributed to the lower solution viscosity, which improves the mobility of the CMC chains. Based on this information, and also on the fact that by using a polymer with a lower molecular mass such as PVP 360, the resulting polymer solution does not have high viscosity. With the loss or decrease of the elasticity of a hydrogel network, the viscous character of the system becomes more pronounced. This behavior can be visualized very well from the values of elastic moduli vs. loss moduli. If the viscous character (G″) is larger than the elastic one (G″ > G′), then it can be assumed that cross-linking has been compromised. As can be seen from Figure 4b, this behavior was not identified for any of the compositions studied.

In the case of the PD10 composition, in the formulation of the hydrogel, in addition to the fact that two natural biopolymers, collagen and chitosan were used, which are more susceptible to degradation during irradiation in solution, PVP with a high molecular weight was also used. We believe that the association between biopolymers susceptible to degradation together with a synthetic polymer with increased molecular mass does not facilitate obtaining a hydrogel with a stable structure, probably due to the reduction of the mobility of macroradicals inside the polymer network. On the other hand, if the molecular weight is increased, the entanglement process will occur. Furthermore, the entanglement process will reduce the movement of macroradicals and the recombination reactions will be suppressed, inevitably leading to the trigger of degradation processes [49]. Considering the above results, the PD81 and PD9 compositions are selected for future studies.

### 3.4. Rheological Analysis

Rheology is a method used to characterize the specific mechanical properties of hydrogels, because changes occurring in the hydrogel structure, such as the degree of cross-linking, and structural and molecular homogeneity/heterogeneity, can be precisely determined. The behavior and magnitude of the moduli (G′ and G″), as well as the flow stress as a function of the applied force, can be mathematically processed, leading to conclusions about the gel structure [50,51].

The values of the G′ and G″ modules can be associated with the cross-linking density (Ve) by using the theory of rubber elasticity, demonstrating that if the elastic modulus of a hydrogel in the swollen state is multiplied by the volume of the polymer (ν_2,S_)^1/3^, the molecular mass between two successive cross-linking points (Mc) can be determined [52,53]. The viscoelastic behavior of the multi-component hydrogels made according to recipes PD81, PD9, and PD10 was investigated by oscillatory rheology in a dynamic regime. The G′ and G′ parameters specific to unirradiated (pre-hydrogels) and e-beam cross-linked hydrogels are shown in Figure 4a,b. Unirradiated polymer mixtures show very low G′ values (around 20–200 Pa) and have a variable behavior depending on the angular frequency. Such rheological behavior is characteristic of viscous fluids with homogeneous structures.

E-beam cross-linked hydrogels showed a considerable increase in G′ modulus compared to that of unirradiated compositions. The G′ modulus values of the cross-linked hydrogels are much higher than those of the G″ modulus (G′ > G″) and are constant over the investigated angular frequency domain.

The gel-like state is highlighted mainly by the elastic characteristic of the entire system, which is predominant compared to the viscous component. Consequently, the G′ moduli of the PD9 and PD81 compositions are several tens of times higher than the loss moduli. In general, the viscoelastic behavior of a given system beyond its gel point, where G′ > G″ and the difference G′-G″, becomes larger as the gel strength increases, is characteristic of physically and chemically cross-linked gels. Similar behavior has been widely described and highlighted in the case of hydrogels based on collagen cross-linked with tannic acid [54]. Consequently, this rheological behavior is characteristic of soft solids such as hydrogels, which have a predominantly elastic and permanent macromolecular network.

PD81 and PD9 hydrogels showed the highest values of G′ between 9697 Pa, and respectively, 14,980 Pa, while PD10 hydrogel has a lower G′ modulus by about an order of magnitude compared to the PD81 composition. The very high value of the G′ modulus specific to the PD9 hydrogel, shows on the one hand the formation of a very strong gel from a structural point of view, but at the same time the formation of a hydrogel with a very rigid structure. These observations are also supported by the lower swelling capacity and the lower mesh size and the increased cross-link density compared to the other two compositions. By comparison, the chitosan-based hydrogel, namely, the PD10 composition, is characterized by G′ approximately two times lower than those corresponding to the compositions PD9, respectively, PD81. In addition to a low G′, the PD10 hydrogel presents a low cross-linking density and a larger Mc parameter. These results highlight a greater contribution of the degradative effects on the component polymers as a result of the effect of ionizing radiation.

On the opposite side, the more cross-linking points are formed inside the hydrogel network, the cross-linking density increases, and the direct consequence of the increase in cross-linking density is reflected by the reduction of the distance between two successive cross-linking points, i.e., the Mc parameter. In our case, the PD81 composition has been prepared without a cross-linker, but the G′ was lower. The G′ value of the PD81 composition was situated as a numerical value between PD9 and PD10. The PD81 composition, besides synthetic polymers, contains CMC and is collagen-rich in ionizable chemical groups. Ionizable groups (H^+^, Na^+^, COO^−^ and NH_4_^+^) belonging to the CMC chain, but also to the collagen, make them susceptible to interaction with other functional groups. Both polymers contain functional groups such as amines and carboxyl which easily form physical or chemical cross-links. Most of the counterions can be electrostatically adsorbed, losing their freedom and giving a more compact and stable network visible by a higher G′ value. Moreover, the interaction between collagen and CMC and other polymer components can be promoted through hydrogen bonding which also can improve structural stability. The higher the concentration of CMC, the conformational stability of collagen can be reduced, due to the stronger electrostatic repulsion between the two macromolecules which have similar functional groups.

An extensive investigation of the rheological properties of hydrogels consists of the comparative analysis of complex moduli (G*). The results are shown in Figure 4c. According to literature data, ideal solids should have a complex modulus equal to the elastic modulus (G* = G′), and an ideal liquid has G* = G″. Thus, G* can be considered as a measure of the stiffness of the material and shows the total resistance of the hydrogels when an appropriate force is applied [55].

The constant variation of the parameters G′, G″, and G* as a function of frequency shows a strong character of the e-beam cross-linked hydrogels, compared to the unirradiated ones. As is shown in Figure 4d, for extended analysis, the experimental data can be evaluated concisely and suggestively employing the loss tangent (tan δ = G″/G′). The dimensionless ratio shows values lower than 1 for the irradiated compositions, compared to the values corresponding to the unirradiated mixtures. Very low values of the loss tangent (lower than 10^−1^) are indicative of hydrogels obtained by irradiation, due to the preponderance of the elastic component. Conversely, for the unirradiated mixtures, the tan δ values were much higher (around 1), a consequence of their viscoelastic behavior.

The results discussed above demonstrate that the use of e-beam processing is a precise and useful method for obtaining mechanically strong hydrogels with different degrees of elasticity, which can be easily handled without losing their structure.

### 3.5. Retention Capability and Water Vapor Transmission Rate (WVTR)

In general, hydrogels designed as wound dressings must exhibit moisture retention to maintain a moist wound environment which allows water vapor transmission, but also to retain their shape when in contact with the wound exudate. From Table 5, it is obvious that multi-component hydrogels retain between 84.8% and 89.2% of moisture after 12 h. R_h_ (%) in 6 h was similar for all the hydrogels, above 90% at room temperature, regardless of their cross-linking density. The R_h_ of the hydrogels was comparable to other PVP/CMC hydrogels which had a value of 70% after about 6 h [14]. Thus, maintaining increased humidity in wounds covered with such a hydrogel provides a beneficial environment for much faster healing.

**Figure 5:**
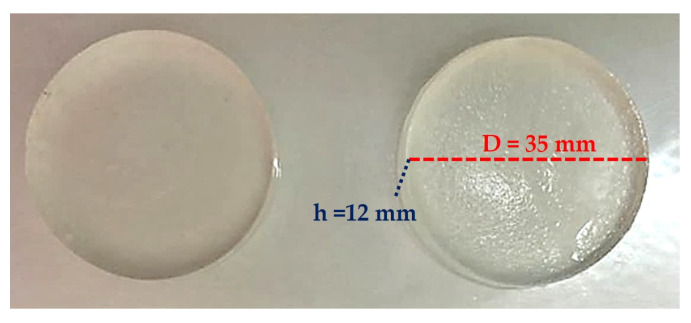
Images of PD81 multi-component hydrogel used in WVTR measurement.

To prevent excessive dehydration, a hydrogel dressing must control water loss from a wound at an optimal rate. Table 6 shows the WVTR values obtained for the studied multi-component hydrogels, irradiated at 25 kGy (Figure 5).

The WVTR values determined for the hydrogels studied were in the range of 53.37–69.1 g/m^2^h, and show that the hydrogels can maintain an adequate moist environment in the wound, reduce wound contraction, and increase tissue regeneration potential, thus accelerating healing wounds. El Salmawi et al. obtained similar results for CS/PVA samples, the WVTR values being between 40–75 g/m^2^h [18]. If the WVTR value is high, it causes the wound to dry quickly, which is not beneficial for the intended application. Moreover, if the WVTR is low, then excess exudate occurs, which slows down the wound healing process and increases the risk of infection.

Bruin et al. have shown that commercial wound dressing (Op Site) with a WVTR of 33 g/m^2^h can cause excess exudate under the hydrogel dressing and lead to infection [56]. Based on the data reported in reference [30], the WVTR of some commercial dressings ranges from 33 g/m^2^h to 208 g/m^2^·h, indicating that the WVTRs of the designed hydrogels in the present study are within a suitable range for wound healing [57].

### 3.6. ATR-FTIR

**Figure 6:**
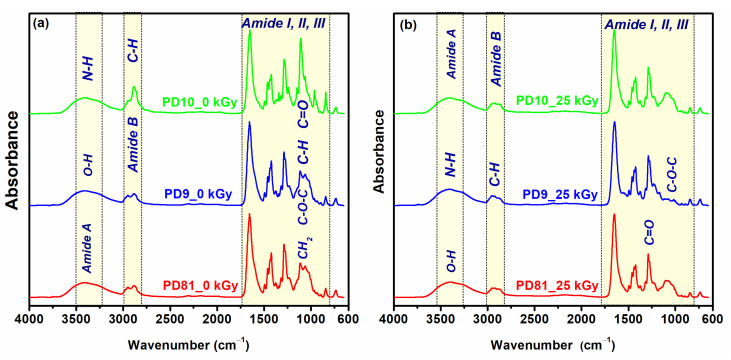
ATR-FTIR of unirradiated (**a**) and cross-linked multi-component hydrogels (**b**)**.**

The e-beam irradiation-induced structural changes in the structure of unirradiated polymer mixture and cross-linked hydrogels were investigated by FTIR spectroscopy. Figure 6a shows the characteristic FTIR spectra of unirradiated and irradiated hydrogels. The unirradiated PD81 hydrogel shows the following specific bands: 3386 cm^−1^ (νOH), 2949–2884 cm^−1^ (CH, CH_2_), 1655 cm^−1^ (amide I, νC=O), 1460/1425 cm^−1^ (−CH_2_ from the pyrrolidine ring), 1281 cm^−1^ (amide III from PVP), 1232 cm^−1^ (amide III from collagen), 1113 cm^−1^ (νC–OH), 1062 cm^−1^ (C–O–C) and 965 cm^−1^ (−CH characteristic of aromatic nuclei in PEO). In the FTIR spectrum for the PD81 hydrogel, it was observed that in the range of 3500–2800 cm^−1^ the absorption bands become broad and increase in intensity. The peak at 3386 cm^−1^ shifted to lower wavenumbers at 3373 cm^−1^ and the peak at 2949 cm^−1^ shifted to 2920 cm^−1^. The characteristic peak of amide I (1650 cm^−1^) shows the same intensity.

The non-irradiated PD9 hydrogel has the main peaks located at: 3395 cm^−1^ (amide A from the collagen structure and assigned to νO–H), 2951/2924 cm^−1^ (νCH, νCH_2_), 1650 cm^−1^ (amide I from collagen, νC=O), 1581 cm^−1^ (amide II from collagen, specific to CN, NH groups), 1435 cm^−1^ (–CH_2_), 1286 cm^−1^ (amide III from PVP), 1228 cm^−1^ (amide III from collagen). At 25 kGy, a shift to higher wave numbers was observed from 2924 cm^−1^ to 2932 cm^−1^ and an increase in the intensity of the absorption bands, due to νCH, CH_2_ and CH3, characteristic of aromatic nuclei. In the FTIR spectrum of the non-irradiated PD10 hydrogel, a broad absorption band is found in the range of 3500–2800 cm^−1^. In this region, the identified peaks were at 3387 cm^−1^ (OH and NH), 2947 and 2881 cm^−1^ (CH from chitosan, collagen, and PVP). Corresponding bands were identified in the range 1700–1000 cm^−1^, both for PVP, chitosan and collagen: 1651 cm^−1^ (amide band I), 1461 cm^−1^ (C=O and CN), 1281 cm^−1^ (amide III), 1061 cm^−1^ (C–O). The decrease in the intensity of the PD10 hydrogel bands at 25 kGy is due to the cross-linking decreases, a behavior also highlighted by the analysis of the hydrogel network parameters. From the FTIR analysis, we appreciated that all the hydrogels produced by e-beam irradiation present the characteristic bands of each polymer included in the recipe, and from a structural point of view, no significant changes were identified between the spectra of the unirradiated polymer mixtures compared to the spectra of the hydrogels obtained after e-beam irradiation.

### 3.7. SEM

**Figure 7:**
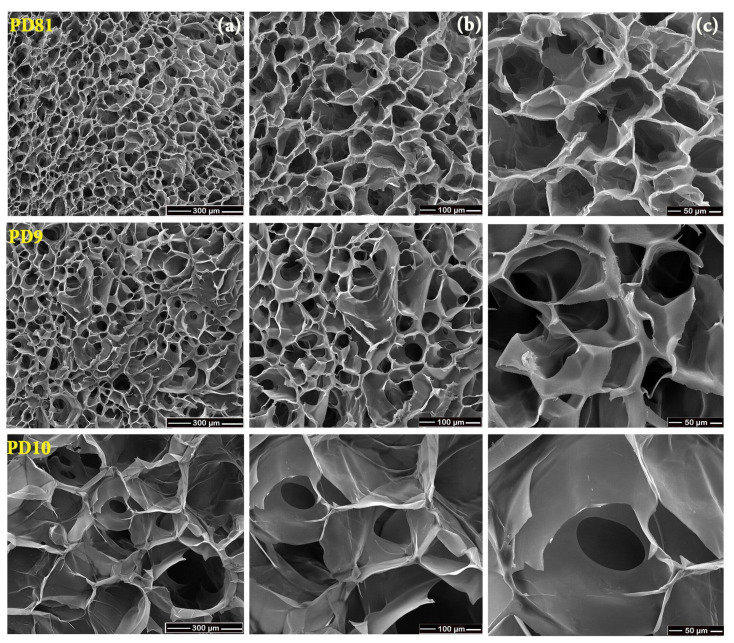
SEM images for PD81, PD9, and PD10 hydrogels: magnification (**a**) 250×; (**b**) 500×; (**c**) 1000×.

The morphological structure of the freeze-dried hydrogels was observed using SEM micrographs acquired at 250×, 500×, and 1000× magnifications and is shown in Figure 7. It is observed that the structure of the multi-component hydrogels varied depending on the composition. The PD81 and PD9 hydrogels present porous structures with pores that are homogeneously distributed which outline a network structure with a 3D conformation. The PD81 hydrogel presents in its structure uniformly distributed micropores with a size between 50–100 µm. As also revealed in the swelling experiments, this structure has better swelling capacity in media with different pH, which also gives a better drug loading capacity to this hydrogel. The PD9 hydrogel, although it exhibits a homogeneously distributed pore structure, at 1000× magnifications a structure with pores smaller than 50 µm, which gives the hydrogel an increased structural rigidity, ultimately leads to a lower swelling capacity. These results correlate very well with the results obtained in the swelling experiments and rheological analysis, which showed that the PD9 hydrogel has a low liquid absorption capacity and increased rheological stiffness. PD10 hydrogel exhibits a microporous structure with inhomogeneous distributed pores that are large compared to the structures of PD81 and PD9 hydrogels, which shows the structurally weaker quality of PD10 hydrogel. The SEM analysis demonstrates that PD10 hydrogel also presented low cross-linking density (0.37) compared to PD81 (1.17) and PD9 (0.78) hydrogels. The low cross-linking density of PD10 hydrogel might be the result of electrostatic repulsion between proton donor-type functional groups such as COOH (arise from acrylic acid) and OH (due to the PEG addition), resulting in a lower mechanical strength.

### 3.8. In Vitro Biological Performance

**Figure 8:**
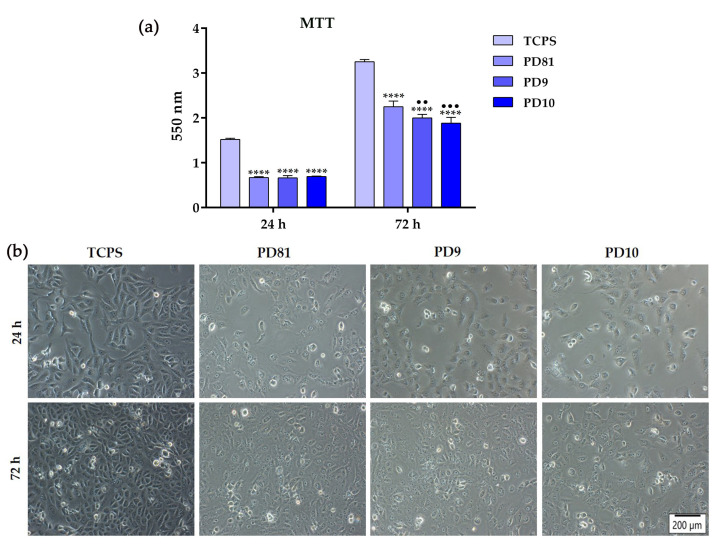
(**a**) The proliferative capacity of Vero cells after 24 h and 72 h of culture in the corresponding extraction media, as evaluated through the MTT assay. Results are presented as means ± SD (n = 3) (**** *p* < 0.0001 vs. TCPS; ●●● *p* < 0.001, ●● *p* < 0.01 vs. PD81); (**b**) Contrast phase microscopical observations of cellular morphology after 24 h cell incubation in the presence of the corresponding extraction media. Scale bar represents 200 µm.

An important condition imposed on every clinical biomaterial is the lack of cytotoxicity; therefore, to determine the possible unfavorable effects exerted by the newly designed hydrogels on the Vero cells’ viability/proliferation, the quantitative colorimetric MTT assay was employed after 24 h and 72 h of cell culture in the corresponding extraction media. As seen in Figure 8a, the time-response study of the total number of metabolically active viable cells revealed a decreasing trend in the proliferative capacity of the cells cultured in the extraction media compared to the control cells grown in the standard medium at any point in time. This being said, it can be observed that at 24 h the cellular viability of the extracts was reduced two-fold (45%) compared to the cells grown in a standard culture medium considered as 100% viability, while at 72 h the viability decreased as follows: 69% and 61% for the PD81 and PD9 extraction media, respectively, and almost two-fold (57%) for the PD10 extract. Moreover, the optical densities (OD) recorded in the case of the cells grown in the corresponding extracts indicated no significant differences between the three analyzed samples, the trend observed only after 24 h of culture. On the contrary, at 72 h after cell seeding, noticeable differences in the number of viable cells between the hydrogels extraction media could be observed, with the PD9 and PD10 extracts showing lower levels of formazan reduction as compared to the PD81 extract. The findings are also confirmed by phase contrast micrographs showing contrasting cellular densities at both experimental time points, suggesting that, to different degrees (Figure 8b), the analyzed extraction media favored cell proliferation without any major deleterious effects. However, even if the extraction media of the newly developed hydrogels proved to hinder cell proliferation, an assessment of which component can be at fault is quite difficult since data found in the literature report on their low toxicity and favorable cell proliferation capacity.

In terms of morphology, the Vero cells cultured in the hydrogels’ extraction media displayed a typically elongated body, with little cytoplasmatic granulation, characteristics similar to those cells incubated in the standard growth medium (Figure 8b), strong evidence that the anchorage-dependent growth and morphological features of cells were not affected by the investigated extracts.

**Figure 9:**
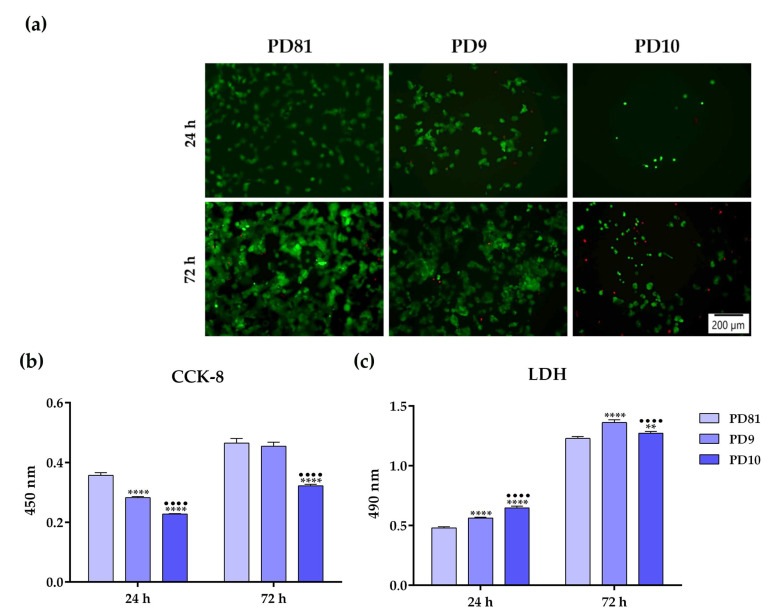
The cytotoxic potential of the newly developed hydrogels, as assessed at 24 h and 72 h after cell seeding directly on the hydrogels’ surface through: (**a**) LIVE/DEAD assay (green fluorescence—living cells; red fluorescence—dead cells); (**b**) CCK-8 test results; (**c**) LDH activity evaluation. Results are presented as means ± SD (n = 3) (**** *p* < 0.0001; ** *p* < 0.01 vs. PD81; ●●●● *p* < 0.0001 vs. PD9). Scale bar represents 200 μm.

Once it was established that the investigated extracts manifested a weak cytotoxic potential, the biological performance of the newly developed hydrogels was further assessed through direct contact studies. For that reason, the cells were seeded directly onto the surface of the analyzed samples, and the impact of these supports on cell viability/proliferation status was investigated through a combination of qualitative and quantitative methods at 24 h and 72 h post-seeding. Thus, the qualitative LIVE/DEAD assay (Figure 9a) revealed significant differences between the developed hydrogels in terms of cell density, a trend observed at both experimental time points. However, no dead red-stained cells could be observed on the surface of the tested supports, except for the PD10 sample, suggesting that the analyzed hydrogels were capable of sustaining cellular survival to a certain degree. In addition, the quantitative CCK-8 assay (Figure 9b) demonstrated that, independently of the incubation period, statistically significant differences between the analyzed samples were observed, with the cells grown on the surface of the PD10 hydrogel exhibiting reduced cellular viability compared to the other two hydrogels, indicating either an inhibitory cellular metabolic activity or a restrictive proliferation capacity. Furthermore, the levels of LDH release into the culture media (Figure 9c) confirm the obtained CCK-8 assay results.

It is a well-known fact that the physicochemical surface characteristics and hydrogel composition play an important role in dictating cellular behavior through specific cell–hydrogel interactions. For example, important processes such as initial cellular attachment, proliferation, differentiation, and new tissue formation are mainly dependent on different scaffold characteristics such as porosity, swelling degree, degradation rate, etc. [58]. Thus, the obtained results can be explained through the PD10 hydrogel unsatisfying physicochemical features such as a non-homogenous structure, a reduced cross-linking density correlated with a low G′ modulus and impaired mechanical stability, characteristics which could negatively impact the initial cellular attachment and cell viability. Moreover, the collagen/chitosan hydrogel exhibited a higher rate of degradation in culture conditions which could lead to a possible restriction of nutrients and metabolites due to the formation of a viscous shell on top of the hydrogel, a phenomenon also reported by Deng et al. in their study [59].

## 4. Conclusions

Three hydrogel recipes (PD81, PD9 and PD10) were developed, whose biological performances were investigated to obtain biocompatible multi-component hydrogels. By evaluating the e-beam irradiation process, it was established that to obtain a cross-linked hydrogel with a stable structure which was sterilized in the same technological step, for the final formulation of the hydrogel, that the optimal irradiation doses are between 20 and 30 kGy. The hydrogels in all three sets are transparent and maintain their structure, are non-adhesive when handling, and most importantly, especially from the point of view of application, they have an elastic structure.

PD81, PD9, and PD10 hydrogels showed different degrees of swelling in deionized water and buffer solutions. The composition PD81 has the highest degree of swelling (1100–3250–1400–1100%) in environments with pH 5.4–6.5–7.4–9.4; in these conditions, the hydrogels are stable and do not dissociate in the swelling environment.

The hydrogels obtained by e-beam cross-linking have a rheological behavior characteristic of soft solids; they present a permanent macromolecular network predominantly elastic. PD81 and PD9 hydrogels showed the highest G′ values between 9697 Pa and 14,980 Pa.

The PD81 hydrogel has the highest cross-linking density (Ve = 1.17 mol/m^3^), which shows the formation of a hydrogel with a dense structure and high stability. The PD10 hydrogel shows a significant decrease in the cross-linking density, also correlated with the decrease in the elastic modulus. These results were suggestive of the selection of compositions PD81 and PD9 for future studies.

Hydrogels maintain between 84.8% and 89.2% of moisture for 12 h. Maintaining increased humidity in infected wounds covered with such a hydrogel provides a beneficial environment for much faster healing.

Morphologically, PD81 and PD9 hydrogels exhibit porous structures with pores that are homogeneously distributed, typical of a network structure with a 3D conformation.

The PD81 hydrogel presents in its structure uniformly distributed micropores with a size between 50–100 µm. This structure has better swelling capacity in environments with different pH, which also gives a better loading capacity for drugs to this hydrogel.

In vitro cell-based studies performed by indirect and direct contact of Vero cells with the developed hydrogels demonstrated that PD9 and especially PD81 samples exhibit the highest degree of biocompatibility. The mentioned hydrogels have an increased potential to be used as drug delivery systems.

## Figures and Tables

**Table 1:** The composition of the three polymer mixtures, expressed per 100 mL of pre-hydrogel.

Pre-Hydrogel Component	Conc. (%)	Hydrogel Code		
		PD81	PD9	PD10
Collagen gel	0.3−0.5	18.18 mL	30.30 mL	30.30 mL
Chitosan	0.5	−	−	0.5 g
PVP 1300	3−3.1	15.5 mL	−	3 g
PVP 360	7	−	7 g	−
PEO	0.2	5.71 mL	−	−
CMCNa	0.625	31.25 mL	−	−
PEG	1	−	−	1 g
MBA	0.25	−	0.25 g	0.25 g
Acrylic acid 0.05 M	3.6	−	−	343 μL
DI−water	92.2−95.8	29.26 mL	62.45 mL	65.20 mL

**Table 2:** Gelation dose (Dg), virtual dose (Dv), and degradation vs. cross-linking degree according to the Charlesby–Rosiak equations.

Sol-Gel Analysis	p_0_/q_0_	Dg (kGy)	Dv (kGy)	R^2^
PD81	0.31	0.44	0.99	0.99
PD9	0.19	0.08	0.52	0.99
PD10	0.12	0.02	3.68	0.99

**Table 3:** Radiochemical cross-linking, G(X), and scission yields, G(S) determined for multi-component hydrogels.

Hydrogel Code	G(X) ^a^/G(S) ^b^ (µmol/J)				
	5 kGy	10 kGy	20 kGy	30 kGy	40 kGy
PD81	0.66 ^a^/0.39 ^b^	0.27 ^a^/0.16 ^b^	0.13 ^a^/0.07 ^b^	0.13 ^a^/0.07 ^b^	0.12 ^a^/0.07 ^b^
PD9	1.78 ^a^/0.67 ^b^	1.28 ^a^/0.48 ^b^	1.07 ^a^/0.40 ^b^	1.00 ^a^/0.38 ^b^	1.10 ^a^/0.41 ^b^
PD10	2.88 ^a^/0.63 ^b^	2.12 ^a^/0.46 ^b^	0.42 ^a^/0.09 ^b^	0.36 ^a^/0.07 ^b^	0.32 ^a^/0.07 ^b^

^a^—G(X); ^b^—G(S).

**Table 4:** The network parameters for PD81, PD9, and PD10 hydrogels.

Hydrogel Code	ρ (kg/m^3^)	G′ (Pa)	$M_{c}$ (kg/mol)	$V_{e}\;\times \;10^{-2}\;(\mathrm{mol}/m^{3})$	ξ (nm)
PD81	1007.8 ± 2.7	9697 ± 24	8.56	1.17	15.8
PD9	1017.6 ± 2.3	14,980 ± 42	13.04	0.78	13.6
PD10	1009.6 ± 5.0	5625 ± 3.1	27.16	0.37	26.7

**Table 5:** Moisture retention capacity at different time intervals.

Time (h)	PD81 (%)	PD9 (%)	PD10 (%)
2	96.8 ± 0.04	96.8 ± 0.08	97.6 ± 0.08
4	93.8 ± 0.06	93.7 ± 0.15	95.2 ± 0.15
6	91.3 ± 0.09	91.1 ± 0.18	93.2 ± 0.20
8	88.6 ± 0.12	88.2 ± 0.18	91.2 ± 0.18
10	86.7 ± 0.12	86.3 ± 0.16	90.0 ± 0.16
12	85.2 ± 0.13	84.8 ± 0.16	89.2 ± 0.13

**Table 6:** Water vapor transmission rate corresponding to hydrogels.

Hydrogel	WVTR (g/m^2^∙h)
PD81	53.37 ± 3.58
PD9	69.10 ± 1.52
PD10	55.04 ± 1.01

## Data Availability

The data presented in this study are available on request from the corresponding author.
